# Constraints on vocal production learning in budgerigars (*Melopsittacus undulates*)

**DOI:** 10.3758/s13420-021-00465-6

**Published:** 2021-03-02

**Authors:** Michael S. Osmanski, Yoshimasa Seki, Robert J. Dooling

**Affiliations:** 1 Department of Biomedical Engineering, Johns Hopkins University, Baltimore, MD 21025 USA; 2 Department of Psychology, Aichi University, Toyohashi, 4418522 Japan; 3 Department of Psychology, University of Maryland, College Park, MD 20742 USA

**Keywords:** Budgerigar, Vocal production learning, Operant control

## Abstract

Budgerigars (*Melopsittacus undulatus*) are small Australian parrots with a well-documented, learned vocal repertoire and a high degree of vocal production learning. These birds live in large, social flocks and they vocally interact with each other in a dynamic, reciprocal manner. We assume that budgerigars must process and integrate a wide variety of sensory stimuli when selecting appropriate vocal responses to conspecifics during vocal interactions, but the relative contributions of these different stimuli to that process are next to impossible to tease apart in a natural context. Here we show that budgerigars, under operant control, can learn to respond to specific stimuli with a specific vocal response. Budgerigars were trained to produce contact calls to a combination of auditory and visual cues. Birds learned to produce specific contact calls to stimuli that differed either in location (visual or auditory) or quality (visual). Interestingly, the birds could not learn to associate different vocal responses with different auditory stimuli coming from the same location. Surprisingly, this was so even when the auditory stimuli and the responses were the same (i.e., the bird’s own contact call). These results show that even in a highly controlled operant context, acoustic cues alone were not sufficient to support vocal production learning in budgerigars. From a different perspective, these results highlight the significant role that social interaction likely plays in vocal production learning so elegantly shown by Irene Pepperberg’s work in parrots.

## Introduction

Social relationships play a critical role in parrot vocal learning (e.g., Farabaugh & Dooling, 1996, Pepperberg, 1999; Wright, 1996; Wright & Dorin, 2001; Wright & Wilkinson, 2000). Nowhere is this more obvious than in the elegant Alex studies by Irene Pepperberg where the performance of “Alex” depends critically on his relationship to his trainer (e.g., Pepperberg, 1993, 1999). There has been a recent resurgence of interest in how widespread the tendency is for vocal production learning across mammals and birds, and to identify the critical components necessary for this behavior (e.g., Brecht et al., 2019; Giret et al., 2012; Martins & Boeckx, 2020; Nieder & Mooney, 2020; Tyack, 2019). One of the criteria, for instance, includes vocalizing in response to an arbitrary stimulus that is temporally contingent and that only occurs in the presence of an instructive stimulus and not in its absence (e.g., Brecht et al., 2019).

Another Psittacine, the Budgerigar (*Melopsittacus undulatus*) is a small Australian parrot that also has a well-documented, learned vocal repertoire (see review in Farabaugh & Dooling, 1996). They live in large, social flocks and interact with each other in a dynamic, reciprocal manner. Vocal learning in these birds occurs during changes in social settings such as the addition and removal of flockmates (Brown et al., 1988; Farabaugh et al., 1994; Hile et al., 2000; Hile & Streidter, 2000; Streidter et al., 2003). Moreover, these birds tend to learn calls from conspecifics that they can see and interact with but not from those they can only hear (e.g., Brittan-Powell et al., 1997; Farabaugh et al., 1994), even though anecdotal and other evidence shows they can learn to mimic sounds from their environment (Gramza, 1970).

Though long a favorite species for hearing studies (Dooling & Saunders, 1975), more recent work has shown that budgerigars can also be trained by operant conditioning to produce different contact calls in response to visual discriminative stimuli such as colored LEDs (Manabe et al., 1995; Manabe & Dooling, 1997). In addition, they can learn, through operant conditioning with selective reinforcement, to modify the pitch and level of their contact calls with a high degree of precision and to produce several different contact calls (Manabe et al., 1997; Osmanski & Dooling, 2009). Moreover, the precision and quality of contact calls produced under operant control is critically dependent on real-time auditory feedback because temporary hearing loss induced from aminoglycoside administration affects the precision of the bird’s call produced under operant control (Dooling et al., 1997). And as the hearing recovers with the regeneration of new hair cells in the inner ear, the precision of vocal production returns (Dooling et al., 1997).

As far as we know, there is no evidence that budgerigars can produce a new contact call upon first exposure. A number of operant studies show that they can be trained to produce new calls by selective reinforcement over a number of days, consisting of one or two 100-trial sessions each day (e.g., Manabe et al., 1997; Osmanski & Dooling, 2009). Considering that these birds in nature, or housed in group conditions, certainly produce thousands of contact calls a day, it seems reasonable to conclude that a bird could learn to imitate a new contact call in the proper social situation in a fairly short amount of time of hours to days.

In this spirit, we recently attempted to train budgerigars to produce two different contact calls to two spatially separated LEDs and to acoustic playbacks of two different contact calls. Results showed birds could use LEDs as discriminative stimuli, producing a different contact call to each of the two LEDs, but could not produce two different calls to two different acoustic playbacks (Seki et al., 2018). This suggests that there are interesting constraints on vocal production learning that call into question what exactly is being learned when budgerigars learn to mimic another bird’s contact call. To that end, here, we further contrast the effectiveness of visual and acoustic stimuli for cuing vocal production in budgerigars.

## Training birds to produce two contact calls under operant control

Following previous work, budgerigar vocal behavior can be brought under basic stimulus-response control in an operant environment, and differential reinforcement using simple sensory stimuli is sufficient to drive vocal learning and plasticity in these birds (Manabe & Dooling, 1997; Manabe et al., 1995; Seki et al., 2018). Here we use differential reinforcement to train birds to produce a particular contact call to visual (LEDs) and/or auditory stimuli (contact calls/tones) that differed either in spatial location (left vs. right) or stimulus features (i.e., visual = color difference, or auditory = either call-type or tone frequency) in order to assess the relative saliency of visual and auditory cues to vocal learning in these birds.

## Methods

### Subjects

The subjects in these experiments were four adult budgerigars from a colony maintained in an aviary at the University of Maryland. Each bird was separately caged and had ad libitum access to water. Because food was used to reinforce vocal behavior, the birds were maintained at 90% of their free-feeding body weight. The University of Maryland Animal Care and Use Committee approved all experimental procedures.

### Apparatus

Birds were trained in an operant testing apparatus consisting of a small wire cage (14 cm × 12 cm × 17 cm) constructed of wire mesh and mounted in an acoustic isolation chamber (Industrial Acoustic Company model AC-1). Three light-emitting diodes (left, center, and right LEDs) were attached to a piece of anechoic foam on the front panel of the cage at approximately the level of the bird’s head. Three small speakers (SONY model MDR-Q22LP) were mounted on the exterior of the cage – one at the center above the front LED panel and one on each of the left and right sides. A small directional microphone (SONY model ECM-77B), located just below the LED panel, detected vocalizations. A food hopper containing hulled millet was located on the floor of the cage under the front LED panel. A small video camera was used to monitor the bird’s behavior while in the chamber.

### Training/testing procedure and analysis

#### Contact call detection and analysis

Custom training/testing and analysis programs were generated using MATLAB software and Tucker Davis Technologies (TDT) System III hardware (Gainesville, FL) and have been published elsewhere (Osmanski & Dooling, 2009; Tu et al., 2011). Briefly, the output of the microphone was amplified, low-pass filtered at 10 kHz, and sent to a circular memory buffer in a TDT real-time digital signal processor (RP2.1) at a nominal sampling rate of 25 kHz. A typical budgerigar contact call has a duration of 100–150 ms and spectral energy concentrated between 2 and 4 kHz (Farabaugh et al., 1998; Farabaugh & Dooling, 1996; Farabaugh et al., 1994). Therefore, incoming signals were classified as a contact call if signal intensity exceeded a user-defined value (because the intensity was different for each bird) for a minimum of 70 ms and if, during this time, the signal power in the frequency band between 2 and 4 kHz exceeded the signal power measured between 4 and 10 kHz.

#### Initial training (shaping)

Birds were first habituated to the experimental chamber and trained to eat from the food hopper when it was activated. Once the birds consistently ate from the raised hopper, manual shaping of vocalizations began. Here, typical aviary sounds were played in the operant chamber to induce the birds to vocalize. Whenever the birds responded to the aviary tape with a contact call, the experimenter activated the hopper. Birds quickly came to associate vocalizing in the test chamber with access to food and, therefore, tape playback was phased out over several training sessions. When birds reliably produced contact calls in the absence of the aviary tape, vocal behavior was reinforced automatically.

Birds were next trained to vocalize only when the center LED was illuminated. Here, the LED turned off each time a vocalization was acquired and turned on again after a random time interval (approximately 5–15 s). Only vocalizations produced when the light was illuminated were reinforced. Vocalizations produced when the LED was turned off caused the random interval timer to reset and increased the wait time before the LED turned back on. Birds successfully completed this phase of training when they reliably vocalized in the chamber both without the flock tape and only in response to the illuminated center LED.

#### Contact call training

Birds were then tested in several further training sessions. Here, the most typical contact call in a bird’s repertoire was selected as that bird’s “template” call (see Manabe & Dooling, 1997). A custom spectral cross-correlation program generated a similarity index between all calls produced in these sessions and these details have been published previously (Osmanski & Dooling, 2009; Tu et al., 2011). Briefly, this program created a spectrogram for each signal using a 256-point Hanning window with 50% window overlap. These spectrograms were then compared using a MATLAB two-dimensional cross-correlation algorithm (MATLAB function XCORR2). This algorithm generated a series of correlation values representing all possible temporal offsets between the two spectrograms. The maximum correlation value was taken as the similarity index between the two calls. This similarity index was then normalized so that it was zero if two calls were perfectly dissimilar and one if the calls were identical. A matrix of similarity values was constructed from all calls produced in a test session and was analyzed using a MATLAB classical multidimensional scaling algorithm. The call in the center of the largest cluster in this two-dimensional space was selected as the template call for the next phase of training.

#### Training in vocal precision with multiple calls

Subsequent training sessions used this template call in order to differentially reinforce vocal behavior. When the center LED was lit, birds were rewarded only for producing calls that were similar to the template call (using the same spectral cross-correlation algorithm described above). Every vocalization produced by a bird was compared to the stored digital template in real-time. The bird was reinforced if the correlation between the two calls exceeded a user-defined value. No reward was given if the correlation did not exceed this value. At first, the criterion was set very low (e.g., r = .01), so that all calls were reinforced. The criterion was then gradually increased over several sessions to a maximum value of r = .70. All training sessions were terminated after 50 reinforcements or 25 min, whichever came first. Subjects were tested in two daily sessions, 5 days per week. All test sessions were separated by at least 3 h.

Once the birds were trained to asymptotic levels of performance on the template-training task described above, a new testing phase was introduced in which budgerigars were trained to produce two different contact calls using a one-back procedure similar to that described by Manabe and colleagues (e.g., Manabe et al., 1997). Briefly, this procedure rewards birds for producing a contact call when an LED is lit that is different from the previously produced call (i.e., the correlation between the two calls does not exceed a user-defined value). Successful completion of this procedure results in birds producing at least two call-types.

## Experiment 1: Relative salience of discriminative stimuli

Once birds were trained to produce two distinct contact calls, they were trained to produce these two calls to different combinations of two complex audio-visual stimuli.

### Subjects

Four adult budgerigars (three males, one female) were used in this experiment and all four birds took part in all conditions.

### Procedure

The two most commonly produced calls from each bird, which were obtained during one-back training (Manabe & Dooling, 1997), were stored as templates and birds were trained to produce a particular call when presented with a specific visual or auditory cue in a series of testing conditions. These conditions were:

A. *Visual discriminative stimulus – location difference.* Cues were compound audio-visual stimuli consisting of two spatially separated (i.e., left and right; 5.0 cm apart) red LEDs and playback of one of the two template calls (i.e., Template A + LED A or Template B + LED B). Call playback occurred through the center speaker above the center panel of the operant cage. Birds were trained to produce a particular call when presented with one of these specific compound stimuli.
B. *Visual discriminative stimulus – quality difference.* Here the cue was a single LED that alternated between the colors red and green. A correct response required a subject to pair a particular color with a specific vocal response. No auditory stimuli were presented in this experiment.
C. *Auditory discriminative stimulus – location difference.* Here the auditory cues were the birds’ own two call templates. Call stimuli were presented through the two speakers located on either side of the operant cage 180° apart. One template was always presented from the right speaker and the other template was always presented from the left speaker. A correct response required a subject to pair a particular acoustic cue (i.e., Template A + Left Speaker or Template B + Right Speaker) with a specific vocal response. Because the acoustic cue is the same call that the bird is required to produce, the bird only needed to repeat what it heard in order to make a correct response. No visual stimuli were presented in this experiment.
D. *Auditory discriminative stimulus – quality difference.* Two different auditory cues (i.e., two tone stimuli (2 kHz and 4 kHz) or two different contact calls) were played through the center speaker above the center panel of the operant cage. A correct response required a subject to pair a particular auditory stimulus with a specific vocal response. In the case of contact call stimuli, the birds’ two stored templates were the auditory stimuli. In other words, the acoustic cues were the exact same calls the birds were required to produce. Thus, they only needed to repeat what they heard in order to make a correct response. No discriminative visual stimuli were presented in this experiment.

Each discriminative cue condition (A–D) required the bird to complete a series of training sessions followed by ten test sessions as follows: Birds were presented with the same stimulus (either visual or auditory and either a quality or a location difference) on successive trials until a correct response was given. Correct responses were followed by a switch to the other stimulus, which was then presented on successive trials until the bird again produced the correct vocalization. Stimulus presentation continued in this manner until a bird’s percent correct for a given session exceeded 80%. At that time, the two stimuli were randomly presented during ten additional test sessions. This random presentation ensured that the animals were attending to the stimulus features when choosing a response and not simply learning to alternate between call-types. Performance was assessed based on the number of sessions required to reach 80% criterion during sequential presentation and percent correct from sessions in which stimulus presentation was random. Each session consisted of 50 reinforced trials (all calls were compared to the subject’s stored call templates), and each trial was composed of one stimulus presentation and one vocal response by the animal.

### Results and discussion for Experiment 1

**Fig. 1:**
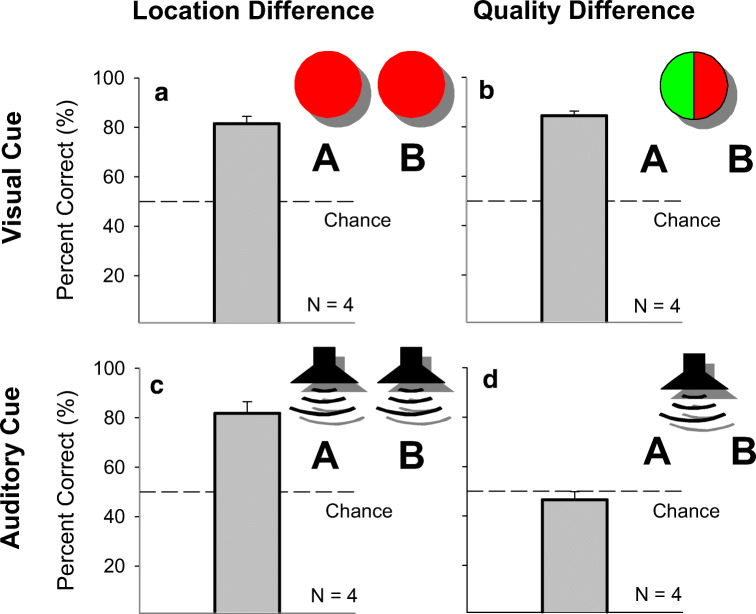
Strength of learning associations between particular contact calls and environmental stimuli in an operant environment. Budgerigars were trained to produce particular contact calls to specific auditory and visual stimuli in order to examine what cues these animals use when making vocal responses. These birds easily learn to associate different calls with location differences among visual stimuli (**Panel A**). Location was manipulated using two LEDs that were spatially separated (e.g., left LED = call-type 1/right LED = call-type 2). Similarly, different calls can be associated with quality differences among visual stimuli (**Panel B**). Here, one centrally located LED was alternated between the colors red and green (e.g., red LED = call-type 1/green LED = call-type 2). Birds were also able to learn associations with acoustic cues differing in location (**Panel C**). Two speakers were hung outside of the testing cage and contact call stimuli were played to the bird (contact call stimulus A = call-type 1/contact call stimulus B = call-type 2). However, birds were unable to learn associations with acoustic cues differing in quality (**Panel D**). Here, either contact call stimuli or tones were played through a single, centrally located speaker (stimulus A = call-type 1/stimulus B = call-type 2)

Results from each of the four discriminative cue conditions were as follows:

A. *Visual stimuli – location difference* (left vs. right red LEDs). All four birds quickly learned to produce a particular contact call to each of the two compound stimuli during sequential trials. The average number of sessions needed to reach criterion was 15 ± 3 sessions. Performance was maintained at or above this level throughout all ten sessions of random stimulus presentation (percent correct: 80.1 ± 1.1 %) (Fig. 1A). These results clearly show that budgerigars can learn to associate different vocalizations with particular audio-visual compound stimuli in an operant environment.
B. *Visual stimuli – quality difference*. All four birds quickly learned to produce the appropriate contact call to each of two visual cues emanating from the same location (green vs. red center LED) during sequential trials. The average number of sessions needed to reach criterion was 11 ± 8 sessions. Performance was maintained at or above this level throughout all ten sessions of random stimulus presentation (percent correct: 84.5 ± 1.9 %) (Fig. 1B). These results show that budgerigars can learn to associate different vocalizations with different visual stimuli originating from the same location in an operant environment.
C. *Auditory stimuli – location difference* (i.e., two speakers). All four birds quickly learned to produce a particular contact call to each of the two auditory cues during sequential trials. The average number of sessions needed to reach criterion was 9 ± 5 sessions. Performance was maintained at or above this level throughout all ten sessions of random stimulus presentation (percent correct: 81.8 ± 4.7 %) (Fig. 1C). These results show that budgerigars can learn to associate different vocalizations with different, spatially separated auditory stimuli in an operant environment.
D. *Auditory stimuli – quality difference* (i.e., one speaker). The subjects were unable to produce a different contact call to different auditory cues coming from the same location (Fig. 1D). Testing was terminated after 28 sessions for all birds after two subjects began producing new, aberrant contact calls. Overall performance remained at chance levels across all sessions (average percent correct: 46.6 ± 3.3%; maximum percent correct across all subjects: 61.4%). Neither contact calls (percent correct: 42.4 ± 5.0%) nor tone stimuli (percent correct: 50.7 ± 5.3%) were sufficient for these birds to make a correct discrimination.

## Experiment 2: Threshold for spatially separated acoustic cues

As a follow-up to the previous experiment, here we tested the minimum spatial separation between sound sources needed to support the production of two learned contact calls.

### Subjects

Three budgerigars (two males, one female) from Experiment 1 were used in this experiment.

### Procedure

In the first phase of this experiment, the relative salience of the auditory component of the audio-visual compound stimuli of Experiment 1A was tested by removing the visual cue once the animals had reached criterion performance. Visual cue removal was accomplished by either turning both LEDs on or turning both LEDs off during stimulus presentation. In other words, birds had to rely solely on the auditory cue in order to produce the correct response. Birds were run in five sessions for each removal condition. Sessions consisted of 50 reinforced trials (all calls were compared to the subject’s stored call template) and each trial was composed of one auditory stimulus and one vocal response by the animal.

In the second phase of this experiment the effect of speaker separation was examined by training the birds on two conditions. Here birds were presented with a playback of either two of their own call templates (as in Experiment 1C) or the call templates of another subject in the experiment. Each subject completed both call stimulus conditions in a pseudorandom order. Birds were required to produce a particular call when presented with a particular playback call in order to obtain food. In the situation where the acoustic stimulus is the same as the call that the bird is required to produce, the bird only needs to repeat what it hears in order to make a correct response. Otherwise, the bird must produce one of their own calls (i.e., one that matches one of their own templates) even though they are presented with another bird’s call.

All stimuli were presented to the birds from one of two small speakers (SONY model MDR-Q22LP), which were hung from a semicircular stand positioned around the front exterior of the testing cage. Speakers were level with the height of the subjects’ head and speaker position was varied along the azimuthal plane of this stand (at 180°, 90°, 45°, 30°, 15°, 10°, 5°, and 0° increments, where 0° was directly in front of the bird). Birds were run in two sessions for each azimuthal position. Sessions consisted of 50 reinforced trials (all calls were compared to the subject’s stored call template) and each trial was composed of one auditory stimulus and one vocal response by the animal.

### Results and discussion for Experiment 2

**Fig. 2:**
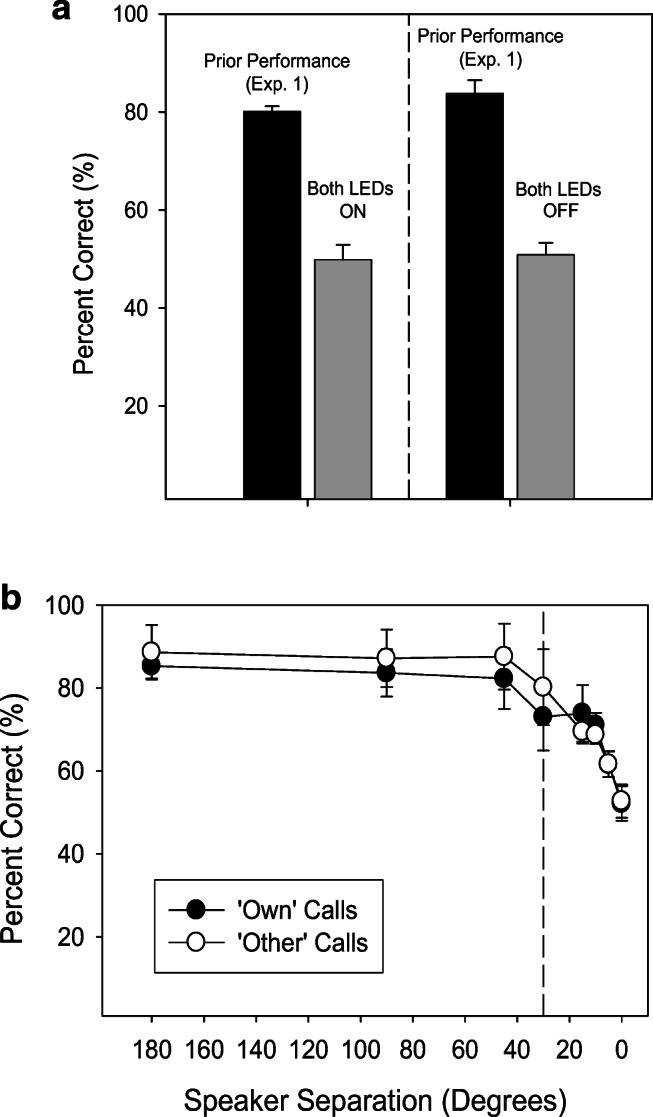
(**A**) Experiment 1A showed that budgerigars can learn to associate vocalizations with different audio-visual compound stimuli. In Experiment 2 (black bars), performance drops to chance levels following removal of the visual stimulus as a response cue (LEDs ON/LEDs OFF). Thus, budgerigars had only learned the visual component of the stimulus when learning the task; the auditory cue was largely ignored. (**B**) Manipulating speaker position restored performance on the learned associations between auditory location cues and vocal responses. The two speakers lose their salience as separate cues at less than about 15–30° separation, which is the typical threshold for discriminating azimuth in budgerigars

In spite of decades of work showing budgerigars easily discriminate among and classify different contact calls (e.g., Dooling et al., 1990), with the visual discriminative cues removed, performance for all three birds dropped to chance levels under both conditions. Average percent correct with both LEDs on was 49.9 ± 3.0 % (Fig. 2A, left panel) and 50.9 ± 2.4 % with both LEDs off (Fig. 2A, right panel). Thus, the birds had learned only the visual cue and failed to attend to the auditory cue when learning the task in Experiment 1A. Interestingly, although the three birds were unable to use an auditory stimulus to guide their choice of call-type, they still used it as a cue to vocalize. That is, subjects withheld vocalizing until they heard the call playback and responded to the auditory cue immediately by producing a contact call in response.

The spatial acoustic cue was effective in supporting performance until the speakers were moved closer together. The performance for all three birds dropped to chance levels as the speaker position was moved from 180° to 0° regardless of whether the birds were presented with playback of their own calls (180° separation = 85.3 ± 3.0 %; 0° separation = 52.9 ± 4.2%) or the calls of another bird (180° separation = 88.6 ± 6.7 %; 0° separation = 52.7 ± 4.0%) (Fig. 2B). In both cases, performance drops below 70% correct as the speaker position is moved to less than 30° separation. This finding is perfectly in line with previous work on measuring the minimum audible angle in budgerigars (Park & Dooling, 1991). In other words, these birds had learned the association between contact call type and speaker position when the speakers are perceptually separated but were unable to use the acoustic differences between their own vocal signals to guide their own vocal production when these two sounds originated from a single source.

## General discussion

The current experiments were designed to examine the control of vocal production learning by external stimuli in budgerigars. We conducted a series of operant experiments in which budgerigars were trained to produce specific contact calls to different auditory and visual cues to further examine whether these birds can respond to specific environmental stimuli with a specific vocal response. Birds learned to produce specific contact calls to different-colored, spatially separated, visual stimuli (Experiment 1A; Fig. 1A), different-colored visual stimuli arising from the same location (Experiment 1B; Fig. 1B), and spatially separated acoustic stimuli (Experiment 1C; Fig. 1C). However, they could not learn to associate a learned vocal response to different acoustic stimuli coming from the same location (Experiment 1D; Fig. 1D) even when the acoustic stimulus and the response were the same (i.e., the bird’s own contact call). This is a rather remarkable finding given prior performance in the suite of tasks.

These operant experiments extend previous findings on vocal production learning in budgerigars, especially the finding that these birds learn calls best in social settings where they can both see and interact with conspecifics (Farabaugh et al., 1994). Experiment 2 is particularly revealing in this regard. While budgerigars do occasionally learn to mimic sounds from their environment (Gramza, 1970), the imitation of a contact call from another bird in a natural setting involves a single contact call coming from a single identifiable sound source (i.e., the bird whose call will be learned). The stimulus configuration in Experiment 2 violates this condition when both calls to be produced are perceived as coming from the same source for the bird (i.e., two speakers separated by less than the minimum audible angle). This is an interesting but perfectly reasonable constraint. And in a general way it also reinforces the need to incorporate interactive social components to fully understand the mechanisms of vocal production learning in this species (e.g., as described in Brittan-Powell et al., 1997; Farabaugh et al., 1994). From the standpoint of classical learning theory, a central tenet has been that any arbitrary stimulus can be associated, through learning, with any arbitrary response, though to be fair, there is now a wealth of information on sensory biases and learning constraints that soften this argument (e.g., Bolles, 1973; Garcia et al., 1974; Hinde & Hinde, 1973).

One of these constraints involves stimulus and response attributes in auditory discrimination tasks. It has long been known that, in general, simple auditory discriminations take significantly longer to learn – up to an order of magnitude longer – using two-choice procedures than the same task takes using go/no-go procedures (Dobrzecka et al., 1966; Lawicka, 1968; for a review, see Burdick, 1979). Miller and colleagues have proposed a general learning principle based on these findings, the “quality-location hypothesis,” which states that stimulus quality (e.g., tone vs. noise) is easily associated with response quality (i.e., go/no-go) and stimulus location (e.g., left vs. right) is easily associated with response location (i.e., go right vs. go left) (Bowe et al., 1987; Miller & Bowe, 1982). However, stimulus quality and stimulus location are not as easily associated with one another.

Sensory biases and learning constraints are known to exist in the vocal production and perception of songbirds and budgerigars (Dooling & Searcy, 1980; Dooling et al.*,* 1990; Marler & Peters, 1977; Nelson & Marler, 1993; Okanoya & Dooling, 1991). It is unclear in the present experiments whether different vocalizations constitute different “qualities” or different “locations” as formulated by Miller and Bowe (1982). However, the present findings in budgerigars nonetheless fit into the more general body of research describing biological constraints on auditory discrimination learning and provide a new limitation on vocal production learning in budgerigars. The failure of these birds to learn an auditory-vocal association, and the ease with which they can learn a visual-vocal association under similar conditions, is remarkable given the overall flexibility in parrot vocal production in general.

The elegant vocal production learning and cognitive “Alex” experiments by Pepperberg and her colleagues (Pepperberg, 1993, 1999) reveal an enormous and complex role for social factors in vocal production learning in parrots. The present operant experiments were purposely designed to not involve these factors and instead to involve repeated stimulus and response pairings that are only controllable in an operant context and that involved recent advances in signal processing algorithms that allow real-time call recognition on a trial-by-trial basis to track learning with a high degree of temporal and acoustic precision. In other words, we attempted to reduce vocal production learning to its simplest components and measure it trial by trial. The results suggest that the elements guiding vocal production learning in budgerigars are still more complicated than we thought. While call learning easily occurs though interactive social facilitation in this species, the current experiments reveal a curious constraint on which stimulus characteristics can support vocal production learning in these birds and perhaps an inability to simply repeat what is heard, at least without social cues. Perhaps what is required is to more closely approximate the conditions in the “Alex” experiments by using facial stimuli, video clips, or live models.
